# Lrp4, a Novel Receptor for Dickkopf 1 and Sclerostin, Is Expressed by Osteoblasts and Regulates Bone Growth and Turnover *In Vivo*

**DOI:** 10.1371/journal.pone.0007930

**Published:** 2009-11-20

**Authors:** Hong Y. Choi, Marco Dieckmann, Joachim Herz, Andreas Niemeier

**Affiliations:** 1 Department of Molecular Genetics, University of Texas Southwestern Medical Center at Dallas, Dallas, Texas, United States of America; 2 Department of Orthopaedics and IBMII: Molecular Cell Biology, University Medical Center Hamburg-Eppendorf, Hamburg, Germany; 3 Institut für Physiologische Chemie und Pathobiochemie, Johannes Gutenberg-Universität Mainz, Mainz, Germany; University Medical Center Groningen, Netherlands

## Abstract

Lrp4 is a multifunctional member of the low density lipoprotein-receptor gene family and a modulator of extracellular cell signaling pathways in development. For example, Lrp4 binds Wise, a secreted Wnt modulator and BMP antagonist. Lrp4 shares structural elements within the extracellular ligand binding domain with Lrp5 and Lrp6, two established Wnt co-receptors with important roles in osteogenesis. Sclerostin is a potent osteocyte secreted inhibitor of bone formation that directly binds Lrp5 and Lrp6 and modulates both BMP and Wnt signaling. The anti-osteogenic effect of sclerostin is thought to be mediated mainly by inhibition of Wnt signaling through Lrp5/6 within osteoblasts. Dickkopf1 (Dkk1) is another potent soluble Wnt inhibitor that binds to Lrp5 and Lrp6, can displace Lrp5-bound sclerostin and is itself regulated by BMPs. In a recent genome-wide association study of bone mineral density a significant modifier locus was detected near the *SOST* gene at 17q21, which encodes sclerostin. In addition, nonsynonymous SNPs in the *LRP4* gene were suggestively associated with bone mineral density. Here we show that Lrp4 is expressed in bone and cultured osteoblasts and binds Dkk1 and sclerostin *in vitro*. MicroCT analysis of Lrp4 deficient mutant mice revealed shortened total femur length, reduced cortical femoral perimeter, and reduced total femur bone mineral content (BMC) and bone mineral density (BMD). Lumbar spine trabecular bone volume per total volume (BV/TV) was significantly reduced in the mutants and the serum and urinary bone turnover markers alkaline phosphatase, osteocalcin and desoxypyridinoline were increased. We conclude that Lrp4 is a novel osteoblast expressed Dkk1 and sclerostin receptor with a physiological role in the regulation of bone growth and turnover, which is likely mediated through its function as an integrator of Wnt and BMP signaling pathways.

## Introduction

Osteoporosis and the inherently increased susceptibility to sustain fractures associated with this disease represent a major challenge in our aging western societies. Bone mineral density (BMD) is used as a reference to diagnose and to monitor osteoporosis and is the best predictor of fractures and a valuable tool for fracture risk assessment [1], [2]. Although much progress has been made in the molecular understanding of bone metabolism in recent years, no therapy is yet available to cure osteoporosis [3]. Many of the current approaches to identify potential therapeutic targets are focused on the Wnt/β-catenin signaling pathway, which is of fundamental importance for osteogenesis [4]–[6]. In a recent genome-wide association study of BMD in individuals of European descent, a new locus was identified near the *SOST* gene at 17q21, which encodes for the osteocyte secreted protein sclerostin [7], a potent inhibitor of bone formation. Within the same study, nonsynonymous SNPs in the *LRP4* gene at 11p11 were suggestively associated with bone density (BMD) [7], confirming previous findings of another study from the same group in which non-significant association of SNPs within or close to the *LRP4* locus with BMD and fractures had been reported [8]. Sclerostin functions as a secreted antagonist of both the bone morphogenetic protein (BMP) [9], [10] and Wnt signaling pathways [11], [12] and Lrp4 has been proposed to function as an integrator of BMP and Wnt signaling [13]. However, whether sclerostin and Lrp4 bind physically to each other, or whether they form an indirect functional interaction has not been known.

Lrp4 is a member of the multifunctional low-density lipoprotein receptor (Lldlr) gene family [14]–[16]. Physiological functions for this ancient gene family include the endocytosis of a large number of macromolecules, including lipoproteins, proteases and protease inhibitors, as well as functions as direct signal transducers or modulators of several fundamental signal transduction pathways, including BMP, TGFβ, PDGF, reelin and canonical Wnt signaling.

Insight into the physiological functions of Lrp4 has been gained through naturally occurring or genetically engineered mutations in mice and cattle. Lrp4 is expressed in various organs [17]–[19], as well as in bone (this study). Mice bearing functional Lrp4 null mutations die perinatally due to a failure of forming neuromuscular junctions [20]–[22]. In addition, limb development is also abnormal [18], [20]. Hypomorphic mutations of the Lrp4 gene are compatible with survival and present with a variable degree of skeletal abnormalities, in particular fusion of digits at the hind and fore limbs (polysyndactyly). By engineering a stop codon just upstream of the transmembrane domain of the murine Lrp4 gene, we have generated such a hypomorphic dysfunctional receptor (Lrp4 *ECD*), in which the lack of a membrane anchor prevents the efficient interaction of Lrp4 with its extracellular ligands. Animals carrying this mutation are viable but present with growth retardation, polysyndactyly and tooth developmental abnormalities [13], [18]. A similar polysyndactyly phenotype has been observed also with other allelic mutations in the murine [23] and bovine [24] Lrp4 gene. It was recently shown that Lrp4 integrates BMP and Wnt signaling during tooth development by binding the BMP antagonist Wise [13]. The role of Lrp4 as an antagonist of canonical Wnt signaling pathway is thought to be mediated in part by a displacement of the homologous Lrp5/6 proteins in the co-receptor complex formed by frizzled proteins (fzd) with Lrp5/6, which is required to bind Wnt proteins and to transduce the Wnt signal to downstream elements of the canonical cascade.

Lrp5/6 are established Wnt co-receptors with important roles in osteogenesis. Gain of function and loss of function mutations in the Lrp5 gene lead to a high bone mass and low bone mass phenotype, respectively, both in mice and humans (for review, see [25]). Mutations in the Lrp6 gene display a partially overlapping bone phenotype with the various Lrp5 mutations (for review, see [26]). Sclerostin is a potent osteocyte secreted inhibitor of bone formation that directly binds to Lrp5 and Lrp6 [12], [27]. The powerful anti-osteogenic effect of sclerostin is thought to be mediated mainly by inhibition of Wnt-signaling through Lrp5/6 within osteoblasts by disrupting the Wnt induced frizzled/Lrp complex formation, although sclerostin was first found to inhibit not Wnt, but the action of murine and human BMPs *in vitro* [9], [10]. Sclerostin is predominantly expressed in skeletal tissues [9], [10]. Mutations in SOST cause the human disease sclerosteosis, which is characterized by massive bone overgrowth [28], [29]. Van Buchem's disease is a similar disorder with generalized hyperostosis and a 52-kb deletion downstream (35 kb) of the *SOST* gene that removes a *SOST*-specific regulatory element [30], [31]. Consistent with a function of sclerostin as an inhibitor of bone formation, transgenic mice overexpressing human *SOST*, display a low bone mass phenotype [10], [32] and *Sost* knockout mice have higher bone mass with increased BMD and bone strength [33]. Interestingly, overexpression of human *SOST* in transgenic mice resulted in an additional phenotype with fused or missing digits of the fore and hind limbs, reminiscent of the phenotype of mice with dysfunctional Lrp4 [32].

Dickkopf1 (Dkk1) is another soluble inhibitor of Wnt/β-catenin signaling that binds to Lrp5 and Lrp6 [34]–[37]. Dkk1 is required for embryonic head and limb development. It also regulates postnatal bone accretion and maintenance of bone mass mainly by binding to Lrp5/6 in a process that involves the transmembrane proteins Kremen 1 or Kremen 2 [34], [38], although at least some of the Wnt-inhibitory effects of Dkk1 mediated by Lrp5/6 seem to be independent of Kremen [39]. Dkk1 null mice die perinatally and show severe developmental phenotypes, including head and limb dysmorphogenesis [40]. A transgenic mouse mutant with reduced Dkk1 expression displays postnatal polysyndactyly, which can be partially rescued by the concomitantly reduced expression of Lrp5/6 [41]. Overexpression of Dkk1 in osteoblasts causes osteopenia [42] and Lrp5 mutants that cannot bind Dkk1 show increased bone mass both in mice and in humans [37], [43]. Dkk1 binds to the first EGF-like domain of Lrp5/6, with which also sclerostin and Wnts interact [34], [44]. It has been shown that Dkk1 can displace sclerostin from the Lrp5 sclerostin complex [45]. Moreover, the expression of Dkk1 has been reported to be regulated by BMPs [46].

The current study was prompted by the association of both the *SOST* and the *LRP4* gene with BMD [7], [8], [47], [48] the established function of both Sost and Lrp4 in the modulation of BMP and Wnt signaling [13], [49], [50], the partially overlapping developmental phenotypes in genetically manipulated mice of the Sost, the Dkk1 and the Lrp4 genes, and our previous findings that Lrp4 binds Wise (a.k.a. Sostdc1) through its extracellular domain which is homologous to that of Lrp5/6 which interacts with sclerostin and Dkk1.

Here, we have used in vitro and in vivo analysis of wild type mice and of two Lrp4 mutant mouse strains, one fully deficient (null mutant, Lrp4-/-; Dietrich et al., in preparation) and a functional hypomorph (Lrp4-ECD; [18]) to show that Lrp4 is an osteoblast expressed receptor for Dkk1 and sclerostin and regulates bone growth and bone turnover *in vivo*.

## Materials and Methods

### Ethics Statement

All animal work has been conducted according to relevant national and international guidelines. In accordance with the recommendations of the Weatherall report, “The use of non-human primates in research.” the following statement to this effect has been included to document the details of animal welfare and steps taken to ameliorate suffering in all work involving non-human primates: This work has been reviewed and approved by the Institutional Animal Care and Use Committee at the University of Texas Southwestern Medical Center (IACUC). This committee functions in close cooperation with the Association for Assessment and Accreditation of Laboratory Animal Care International (AAALAC). The performance site for this work (UT Southwestern) is an AAALAC accredited institution.

### Production of Recombinant Proteins and Binding Assay

HEK293A cells were transfected with pCDNA3-AP, pCDNA3-RAP-AP, pCDNA3-Wise-AP, pCDNA3-RSpondin2-AP, pCDNA3-DKK1-AP, or pCDNA3-SOST-AP constructs using FuGENE 6 (Roche) in DMEM plus 0.2% BSA medium to produce conditioned media containing AP or AP-tagged recombinant proteins. After 48 h transfection, media were collected and the production levels of AP and AP-tagged proteins in the media were determined by AP activity assay using p-Nitrophenyl phosphate (Calbiochem) as a substrate. To investigate the binding of AP-tagged proteins to Lrp4 in a cell-free system, media containing of the Lrp4 ectodomain fused to the constant region of Immunoglobulin G (Lrp4ecto-Fc) was produced by transfection of HEK293A cells with pcDNA3-LRP4ecto-Fc in DMEM plus 0.2% BSA medium. The medium containing LRP4ecto-Fc was incubated with Protein A-Agarose beads (Sigma) at 4°C for 2 h to bind LRP4ecto to the beads. Equal volumes of media containing AP or AP-tagged proteins were pre-cleared with Protein A-Agarose beads at 4°C for 2 h and then incubated with the LRP4ecto-bead conjugates at 4°C overnight. The conjugates were washed four times with PBS, resuspended in 40 µl Laemmli sample buffer, and western blotting was performed using anti-AP antibody (Sigma). To investigate binding of AP-tagged proteins to LRP4 in cell system, HEK293A cells were transfected with pCDNA3-LRP4 full length constructs for 48h, washed once with PBS plus 0.1% BSA, and incubated in equal volumes of media containing AP or AP-tagged proteins at 37°C for 1 h. The cells were washed once with PBS, incubated with the cross-linker dithiobis[succinimidylpropionate] (250 µM, Pierce), at room temperature for 30 min, harvested, washed three times with PBS, and lysed in 50 mM Tris-HCl buffer, pH 7.5 containing 150 mM NaCl, 1 mM MgCl_2_, 1 mM CaCl_2_, 1% Triton X-100, and protease inhibitors (Roche). After determination of protein concentration by Lowry assay, equal protein amounts of the cell lysates were subjected to immunoprecipitation using anti-AP antibody and Protein A-Agarose beads. Immunoprecipitates were boiled in SDS sample buffer containing 5% β-mercaptoethanol and western blotting was performed using anti-Lrp4 antibodies derived against the carboxy terminus and the murine Lrp4 ectodomain, respectively.

### Animals

Lrp4ECD mice were generated by introducing a stop codon into the exon 36 of *Lrp4* gene, resulting in the extracellular truncation of the protein and the loss of the transmembrane and intracellular domains of LRP4 [18]. To minimize the effects of genetic drift and vertical inbreeding, mice from the entire Lrp4ECD heterozygote pool of our colony on a 129SvEv x C57BL/6 hybrid background were crossed to produce wild type and Lrp4ECD littermate controls. All animals were maintained in the UT Southwestern animal facility with 12h light/12 h dark cycle and fed a standard rodent chow diet (Diet 7001, Harlan Teklad, Madison, WI) with access to water *ad libitum*. At ten weeks, experimental mice were fasted for 4 h prior to collection of urine. After urine collection, the mice were euthanized with isoflurane prior to collection of blood followed by harvesting of the calvaria, lumbar spine, and femur. All procedures were performed in accordance with the protocols approved by the Institutional Committee for Use and Care of Laboratory Animals of the University of Texas Southwestern Medical Center at Dallas (IACUC).

### Determination of LRP4 Expression in Bones and Osteoblasts

Calvaria and femur were cleaned by removing soft tissues connected with scalpel and snap-frozen in liquid nitrogen for storage until the extraction of protein and RNA. Primary osteoblasts were obtained by sequential collagenase digestion of calvariae from 3–4 day old wildtype C57Bl/6 mice as described [52].Osteoblast differentiation was induced at 80% confluency in αMEM containing 10% fetal bovine serum, 50 µg/ml ascorbic acid and 10 mM β-glyerophosphate. At day six of differentiation, total cell proteins were isolated and subject to western blot analysis. To extract bone proteins, calvaria and femur were ground in a mortar in liquid nitrogen followed by sonication in 50 mM Tris-HCl buffer, pH 7.5 containing 150 mM NaCl, 1 mM MgCl_2_, 1 mM CaCl_2_, 1% Triton X-100, and protease inhibitors (Roche). After determination of protein concentration according to Lowry, the extracted bone proteins were subjected to western blotting with anti-LRP4 antibodies along with brain proteins from wildtype and from *Lrp4* null mice as positive and negative controls, respectively.

Total RNA from calvaria and femur was extracted in RNA STAT-60 (Tel-Test Inc.) using a Polytron homogenizer. The RNA was treated with DNase I (Ambion Inc.) to exclude potential contamination by DNA. Two µg of DNA-free RNA were reverse-transcribed using TaqMan Reverse Transcription Reagents (Applied Biosystems). Quantitative real-time PCR analysis was performed using the ABI PRISM 7900HT Sequence Detection System (Applied Biosystems). Each 20 µl reaction contained 20 ng/µl cDNA, 2.5 µM forward and reverse primers, and 10 µl of 2x SYBR Green PCR Mater Mix (Applied Biosystems). All samples were run in triplicates and specific amplification of a single PCR product with the expected length was confirmed with gel electrophoresis. The following primers were used:

LRP4, 5′-TCTGCGCACACGGAATAGC (forward),

5′-GCGCTCACCGCACATGT (reverse);

36B4, 5′-CACTGGTCTAGGACCCGAGAAG (forward),

5′-GGTGCCTCTGGAGATTTTCG (reverse).

### μCT analysis of Bone

The femoral and spinal bones of mouse were scanned at an isotropic voxel size of 27 µm (80 kV, 450 µA and 2000 ms integration time) using the eXplore Locus micro-CT scanner (GE Health Care). The scan was done transversely which generated 720 tomographic slices from a 33 mm sample depth. Three dimensional images were then reconstructed from these two-dimensional gray-scale image slices using the reconstruction utility (GE Medical System) and visualized using Microview Software (GE Medical System). Density values for soft tissue and bone were calibrated using a phantom (GE Health Care) containing air bubble, water and hydroxyl apatite rod. The region of interest (ROI) for each animal tissue was defined based on skeletal landmarks from the grey-scale images.

Bone analysis was conducted using Microview software. For femoral analysis, bone mineral density (BMD) and bone mineral content (BMC) were measured in cortical bones. For determination of cortical bone, an appropriate ROI (region of interest) was defined and the histogram was created on this selected region. The separation of cortical region was calculated from the grey scale values (upper threshold 4300 and lower threshold 900) on the histogram. BMD, BMC and trabecular structural measurement in vertebral bone was done by Microview using the threshold selected for trabecular bone (upper threshold 1400 and lower threshold 600). Data analysis and calculations were automatically performed by the software. All μCT analyses were done with samples in a randomly selected sequence by an independent blinded investigator who did not know the study hypothesis.

### Measurement of Bone Formation and Resorption Markers

All serum and urine samples had been stored at minus 70°C immediately after the blood draw and were not unfrozen prior to analysis. Serum levels of osteocalcin were measured using a two-site immunoradiometric assay (Immutopics) according to the manufacturer's instructions. Alkaline phosphatase activity in serum, was measured using the pNPP method. Briefly, 5 µl of serum were mixed with 50 µl of substrate solution containing 2 mg/ml of p-Nitrophenyl Phosphate (Sigma) in alkaline buffer solution (Sigma). After incubation at 37°C for 15 min, the reaction was stopped by adding 500 µl of 0.5M NaOH and the absorbance at 405 nm was measured. Alkaline phosphatase activity was determined by using p-nitrophenol solution (Sigma) as standard substrate.

Bone resorption was determined by measuring urinary levels of deoxypyridinoline (DPD) using MicroVue DPD (Quidel) according to the manufacturer's instructions. The DPD values were normalized to the levels of urinary creatinine measured using the MicroVue Creatinine Assay Kit (Quidel).

## Results

### Lrp4 Interacts with Dkk1 and Sclerostin *In Vitro*

**Figure 1:**
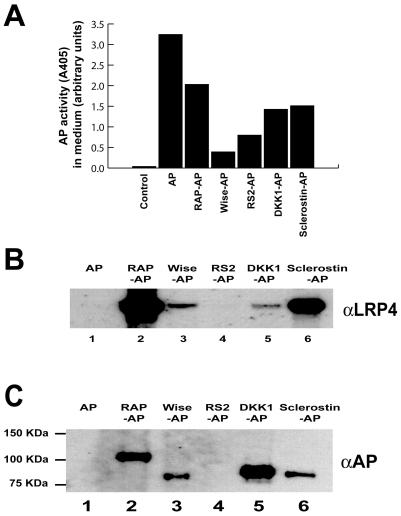
Dkk1 and sclerostin bind to Lrp4. *Panel A*, HEK293A cells were transfected with alkaline phosphatase (AP) as a control and with AP-tagged confirmed and putative Lrp4 ligands. The relative abundance of the respective ligands in the cell culture supernatant is shown as AP activity (arbitrary units). *Panel B*, Conditioned media containing the indicated AP-fusion proteins were incubated with HEK293A cells overexpressing Lrp4. After binding and crosslinking, AP-fusion proteins were immunoprecipitated, and coprecipitated LRP4 was detected by immunoblotting. RAP constitutively interacts with LDL receptor family members and serves as a positive control. R-spondin2 served as a negative control. LRP4 coprecipitates with Dkk1 and sclerostin AP-fusion proteins. *Panel C*, In a cell free assay, soluble Lrp4 ectodomain-Fc fusion protein was conjugated to Protein A-Agarose, incubated with media containing the AP-tagged putative Lrp4 ligands and Lrp4 bound ligands were then detected by immunoblotting against AP.

Both Dkk1 and sclerostin modulate Wnt signaling by binding to EGF-like repeats of Lrp5 and Lrp6. The extracellular domains of Lrp5, Lrp6 and Lrp4 are highly conserved [18], raising the possibility that the spectrum of ligands that bind to Lrp5/6 and Lrp4 might overlap. In order to test for a physical interaction between Dkk1 and Lrp4 as well as sclerostin and Lrp4, we performed two types of *in vitro* binding assays. First, HEK293A cells transfected with Lrp4 were incubated with conditioned media containing the alkaline phosphatase (AP)-tagged putative Lrp4 ligands (Figure 1A). The 39kDa receptor-associated protein (RAP), a chaperone for Ldlr gene family members, is known to bind to Lrp4 [51]. We have previously shown that Wise interacts with Lrp4, while another modulator of the Wnt signalling pathway, R-spondin2 (RS2) does not [13]. Thus, RAP and Wise served as positive controls, RS2 and non-tagged AP as negative controls for these assays. AP-fusion proteins of controls and the putative ligands Dkk1 and sclerostin were pulled down with an antibody against AP and immunoblotted with an anti-Lrp4 antibody to detect co-precipitated receptor (Figure 1B). In a complementary, converse cell free assay, the Lrp4-Fc fusion protein was immobilized on Protein A-agarose and incubated with the AP-tagged putative ligands and controls. Lrp4 bound ligands were then detected by immunoblotting against AP (Figure 1C). Both assays revealed that Lrp4 efficiently binds Dkk1 and sclerostin.

### Lrp4 is Expressed in Osteoblasts

**Figure 2:**
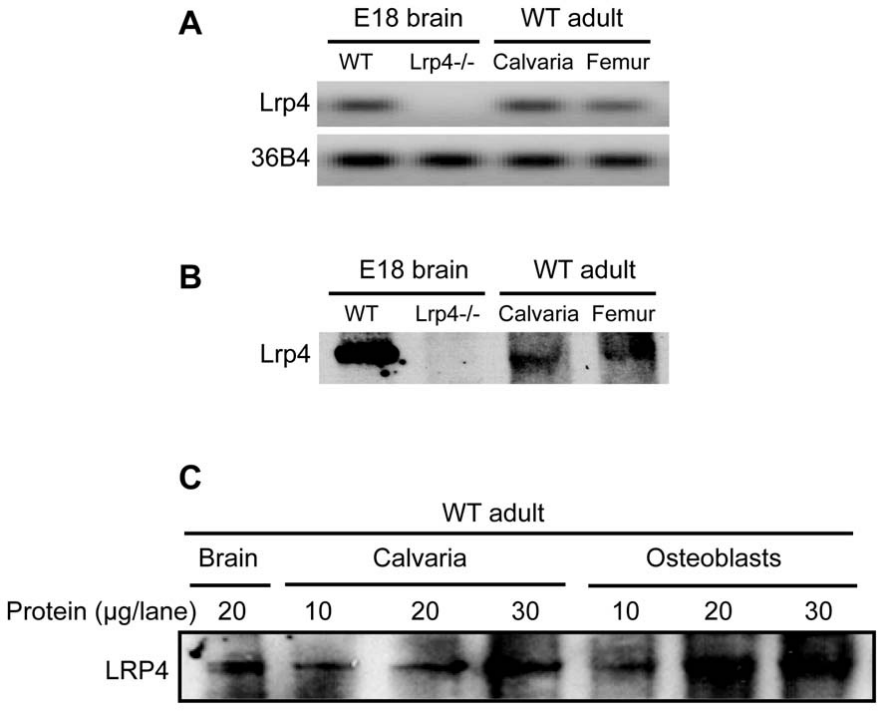
Lrp4 is expressed in bone. *Panel A*, Agarose gel of the PCR products from the experiment shown in the Table. *Panel B*, Extracted proteins were subjected to immunoblotting with an antibody against LRP4. *Panel C*, Protein extracts from primary osteoblast cultures after 6 days of in vitro differentiation were subjected to anti-LRP4 immunoblotting. Brain and calvarial extracts are shown as controls.

Sclerostin is a secreted protein expressed by osteocytes. Both sclerostin and Dkk1 bind to Lrp5/6, which play critical roles in the Wnt/β-catenin signal transduction in osteoblasts. The inhibition of bone formation by Dkk1 and sclerostin is thought to be mediated primarily through inhibition of wnt-signaling in osteoblasts. In light of our finding that Dkk1 and sclerostin bind to Lrp4 (Figure 1), we next analyzed whether Lrp4 is expressed in bone. Figure 2A-C and Table 1 demonstrate by quantitative RT-PCR and Western blot analysis that Lrp4 mRNA and protein are indeed expressed in calvaria and in the long bones of adult wild type mice. Murine embryonic brain (E18) from Lrp4 null mice and wild type littermates served as a negative and positive control for the RT-PCR and immunoblots, respectively (Figure 2A, B). Moreover, a polyclonal antibody derived against the recombinantly expressed Lrp4 ligand binding domain specifically recognizes Lrp4 in brain protein extracts (Figure 2B). In Lrp4 null animals, in which the promoter and exon 1 of Lrp4 have been deleted (Dietrich et al., in preparation) neither mRNA (Figure 2A, Table 1), nor Lrp4 protein was detectable (Figure 2B), whereas mRNA and protein were present in wild type calvaria, femur (Figure 2A, B) and primary calvarial osteoblasts from newborn wild type mice that had undergone six days of *in vitro* differen>tiation with ascorbate and β-glycerolphosphate (Figure 2C). Taken together, these data demonstrate that Lrp4 is expressed by osteoblasts in murine bone.

**Table 1:** Lrp4 is expressed in bone.

Gene	WT E18 Brain Ct (avg.±±S.D.)	Lrp4-/- E18 Brain Ct (avg.±±S.D.)	WT adult calvaria Ct (avg.±±S.D.)	WT adult femur Ct (avg.±±S.D.)
Lrp4	27.9±0.06	N.D.	28.8±0.10	31.3±0.21
36B4	20.7±0.02	21.0±0.01	24.4±0.01	25.9±0.03

Total RNA was extracted from embryonic brain (E18), calvaria and femur cortical bone of 10-week-old adult mice. Quantification of Lrp4 and control (36B4) mRNA was performed by real-time PCR. Results represent the average of cycle threshold (Ct)±S.D. in triplicate.

### Functional Lrp4 Deficiency Results in Impaired Skeletal Growth, Reduced Trabecular Bone Volume and Increased Bone Turnover

**Figure 3:**
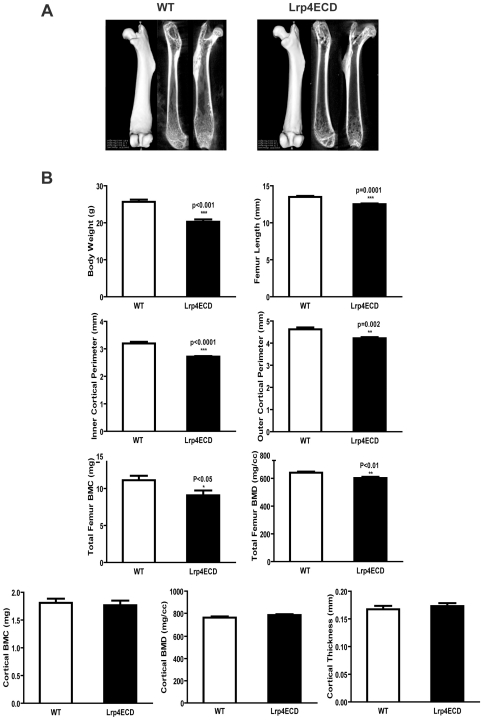
Lrp4ECD results in impaired bone growth. *Panel A*, Representative μCT images of WT and Lrp4ECD mouse femurs. *Panel B*, Total body weight of ten week old male mice was determined prior to sacrifice. Spinal column and femurs were dissected out, fixed in 4% paraformaldehyde and scanned. Calculations for the indicated parameters were performed using Microview software from GE Medical System. Results represent the average±S.E. of values from six male mice.

**Figure 4:**
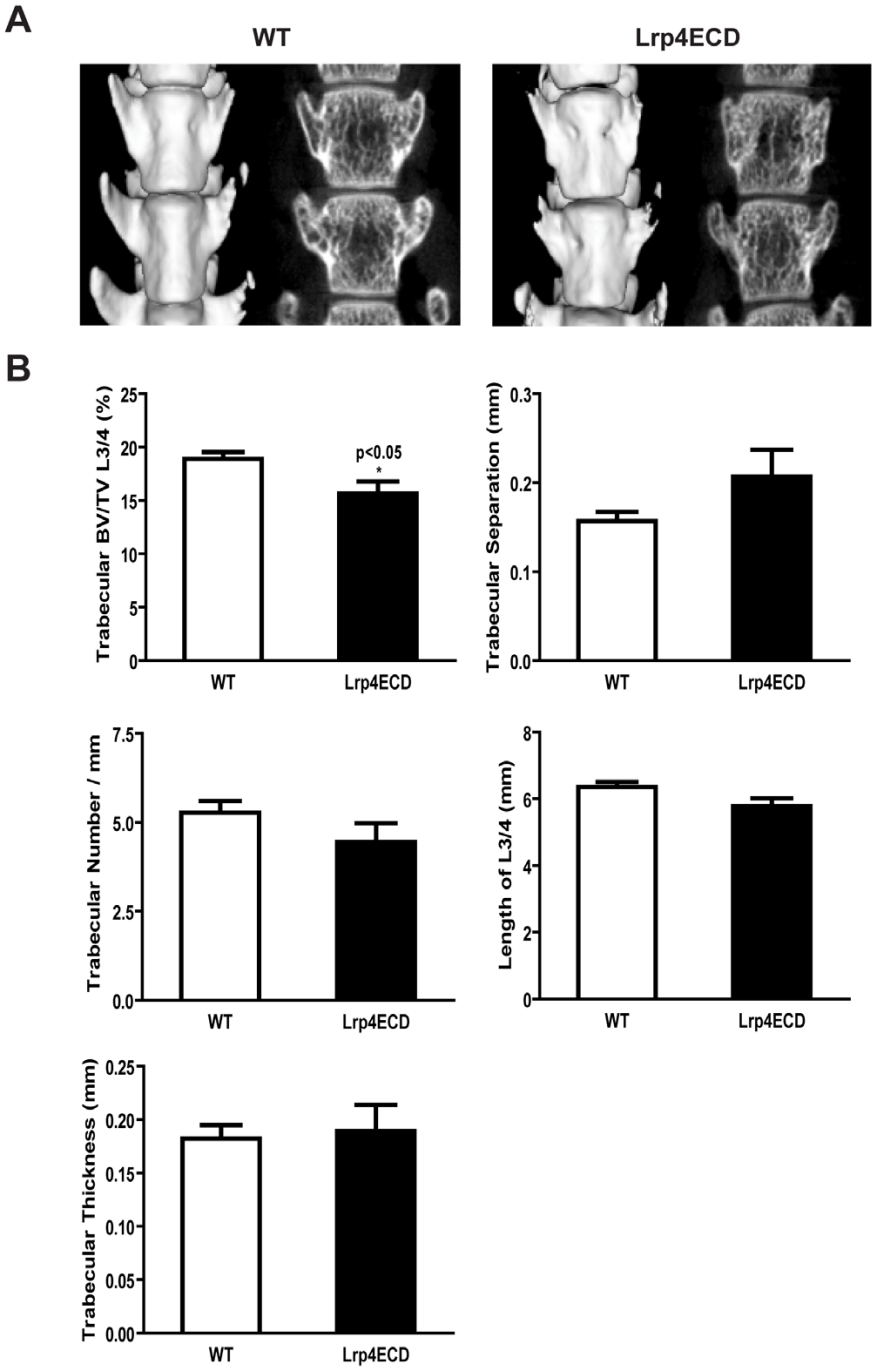
Lumbar spine trabecular bone is reduced in Lrp4ECD mice. *Panel A*, Representative μCT images of wild type and LRP4ECD mouse lumbar vertebra L3 and L4. Panel B, Lumbar vertebrae were fixed, scanned, and analyzed for the indicated parameters. Results represent the average±S.E. of values from six male mice.

To investigate the physiological significance of the expression of Lrp4 by osteoblasts, we performed micro computer tomography (μCT) of the femur and lumbar spine vertebrae of Lrp4ECD mutant mice and wild type littermates. We also measured several biochemical markers that are indicative of bone turnover. Ten week old male animals were analyzed (n = 6 per genotype for μCT, and n = 14 per genotype for the determination of turnover markers). Fasting blood and urine samples were obtained before sacrifice according to a standardized time schedule to rule out any potentially confounding circadian alterations in the concentrations of the osteoblast activity markers alkaline phosphatase (ALP) and osteocalcin (OCN) as well as the urinary collagen breakdown product desoxypyridinoline (DPD) as a marker of osteoclast activity. Body weight of the animals was determined upon sacrifice. Ten week old wildtype mice had a significantly higher total body weight (25.7 +/− 1.3 g) than their Lrp4ECD littermates (20.3 +/− 1.4 g) (Figure 3B), consistent with our earlier findings [18]. Polysyndactly of fore limbs and hind limbs and abnormal tooth development are fully penetrant [18]. MicroCT 3D and 2D reconstruction of the femur did not reveal striking differences in the overall morphology of the bones (Figure 3A), but showed that the reduction of total body weight was accompanied by a significant reduction of femur length (13.5 +/− 0.3 mm vs 12.5 +/− 0.2 mm p = 0.0001) and a clear, albeit statistically non-significant trend towards a reduction in the combined height of lumbar vertebrae L3 and L4 (6.36 +/− 0.36 mm vs 5.78 +/− 0.58) of Lrp4ECD mutants as compared to controls (Figure 3B and 4B). Furthermore, the inner and outer cortical perimeter of the femur mid-diaphyses was significantly smaller in Lrp4ECD mutants (Figure 3B, p<0.0001 and p = 0.002). These findings indicate that at least part of the reduction of the total body weight was due to impaired skeletal growth. This is consistent with our previous report of a role of Lrp4 as a modulator of the BMP and Wnt signaling cascades in limb and tooth development, since both pathways also play a role in the enchondral ossification process in the growth plate that controls limb length growth. Total femur bone mineral content (BMC) and bone mineral density (BMD) were also significantly reduced in Lrp4ECD mutants (BMC 11.1 +/−1.3 mg versus 9.0 +/− 1.5 mg, p<0.05 and BMD 641.3 +/− 16.7 mg/cc versus 602.3 +/− 21.4 mg/cc, p<0.01), while cortical thickness, cortical BMC and cortical BMD were unaffected (Figure 3B), suggesting that the difference in total femur BMD/BMC was due to differences in the trabecular compartment of the femur. For the assessment of trabecular bone structure and density in mice, the lumbar spine is better suited than the distal femur or the proximal tibia. We therefore performed μCT on the lumbar vertebrae L3 and L4. Again, 3D and 2D reconstructions did not unveil gross abnormalities of lumbar spine morphology (Figure 4A), but trabecular bone analyses revealed a significantly reduced bone volume per total volume (BV/TV, 18.9 +/− 1.5% vs 15.7 +/− 2.5%) in Lrp4 ECD mutants as compared to wild type littermates, accompanied by the according changes in trabecular separation and trabecular number (Figure 4B). Trabecular thickness was not significantly affected (Figure 4B). Taken together, μCT analyses of the femur and lumbar spine indicate impaired bone growth in Lrp4ECD mutant mice, with smaller bones of otherwise mostly normal morphology and suggest a concomitant relative increase of bone resorption over bone formation, resulting in a net loss of trabecular bone.

**Figure 5:**
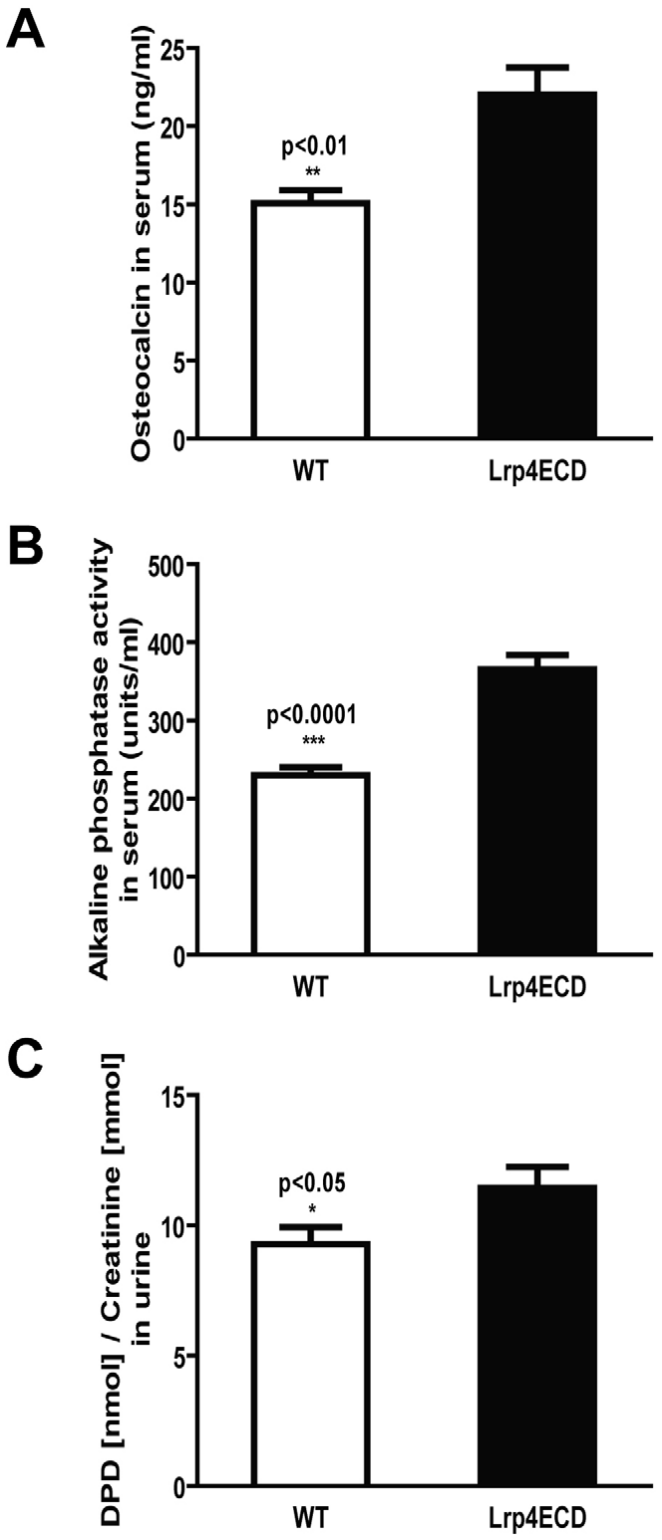
Bone turnover markers are increased in Lrp4ECD. Urine and blood samples were collected from the same mice that were used for analyses in Figure 3 and 4. *Panel A*, The bone formation markers osteocalcin and alkaline phosphatase were determined in serum. (B) Urinary levels of DPD/creatinine were measured as a marker for bone resorption.

To further explore the underlying cause of this phenotype, we measured the osteoblast activity markers osteocalcin (OCN, Figure 5A) and alkaline phosphatase (ALP, Figure 5B) in serum and urinary deoxypyridinoline (DPD) as an osteoclast activity marker (Figure 5C). All parameters were significantly elevated in the Lrp4ECD mutants (OCN: 15.1 +/−1.9 ng/ml vs 22.0 +/− 3.9 ng/ml; ALP: 230.2 +/− 22.3 units/ml vs 365.5 +/− 41.1 units/ml; DPD/creatinine: 9.3 +/− 1.9 nmol/mmol vs 11.4 +/− 2.4 nmol/mmol), indicating increased bone turnover in comparison to wild type control animals.

## Discussion

Here we report that Lrp4 binds the two secreted Wnt modulators Dkk1 and sclerostin, that Lrp4 is expressed by murine bone, specifically by osteoblasts, and that mice carrying a functionally hypomorphic Lrp4 mutation have impaired bone growth and increased bone turnover.

Canonical Wnt-signaling is of crucial importance for osteogenesis and has probably been the most intensely studied signaling pathway in bone in recent years. Lrp5 and Lrp6 have been recognized as the main osteoblast expressed co-receptors of the frizzled family of proteins, which together form a receptor complex at the cell surface that binds the Wnt signaling proteins and thereby initiates the canonical signaling cascade. The finding that Lrp4 is also expressed by osteoblasts in bone has important implications for the understanding of the complexity of the regulation of the canonical Wnt signaling pathway in bone. Sclerostin and Dkk1 are inhibitory modulators of the Wnt/β-catenin pathway and it has been generally assumed that these molecules exert their antagonistic effect mainly through direct binding to Lrp5/6, thereby blocking binding sites on these receptors for the pathway activating Wnts. Here we have shown that Lrp4 efficiently binds both sclerostin and Dkk1, suggesting that at the osteoblast surface, Lrp4 could either compete with Lrp5/6 for the interaction with sclerostin and Dkk1 or cooperate with the Lrp5/6/frizzled co-receptor complex by binding and presenting these ligands to Lrp5/6. LRP4, but not Lrp5/6, contains an NPXY endocytosis motif in its cytoplasmic domain. However, it is not known whether Lrp4 contributes to the endocytosis of extracellular molecules to a significant extent. The exact nature of both the cellular consequences of sclerostin and Dkk1 binding to Lrp4 as well as the impact that this process may have on the signaling function of Lrp5/6 in bone and other tissues will have to be determined by future studies. It is likely that mouse genetics with double and triple knockout combinations of receptor and ligand genes will be needed to fully understand these interactions.

How can we explain the bone phenotype of Lrp4ECD mice? We have previously shown that Lrp4 integrates BMP and Wnt signals in tooth development in mice [13] by binding Wise, a secreted BMP antagonist that also binds to Lrp6 and thereby inhibits Wnt signaling. In analogy to Wise, sclerostin and Dkk1 are two other Lrp5/6 ligands that we have now shown to also interact with Lrp4. One possible mechanism is that Lrp4 simply acts as a sink and competes with Lrp5/6 for the binding of these Wnt antagonists, which then are no longer available for suppression of the signal through the Lrp5/6 axis. When Lrp4 is dysfunctional as in the Lrp4ECD mutant strain that we used here, the availability of extracellular Wnt- antagonists increases, resulting in a net inhibitory effect on canonical Wnt/β-catenin signaling in the cell, which is well known to decrease BMD and thus would provide an explanation for the reduced lumbar spine trabecular BV/TV in the Lrp4ECD mutant mice. Furthermore, both Wnt signaling and BMP signaling are involved in the growth plate organization and enchondral ossification process [53]–[56]. Moreover, Lrp4 can by itself inhibit Wnt signaling, presumably by competing for Lrp5/6 in the Wnt/Fz complex [18]. Since sclerostin can inhibit BMPs, and Dkk1 is itself regulated by BMPs, it is likely that the failure to properly integrate BMP and Wnt signaling pathways in the absence of a normal, functional Lrp4 is responsible for the reduced limb length growth in the Lrp4 ECD mutants. Such a mechanism would be analogous to the role of LRP1 in the integration of PDGF and TGFβ signals in the vascular wall, where loss of LRP1 expression in smooth muscle cells results in the simultaneous deregulation of PDGF as well as TGFβ signalling with medial hypertrophy, elastolysis and fibrosis [57]–[59].

The elevated concentration of biochemical bone turnover markers, serum osteocalcin, alkaline phosphatase and urinary DPD in Lrp4ECD animals indicate that both osteoblast and osteoclast activities are significantly increased in these mice. This could be either explained by an altered coupling of osteoblast and osteoclast activity due to osteoblast-specific changes in Wnt and BMP signaling, or by altered osteoclast function independent of osteoblasts. We failed to detect Lrp4 expression in primary osteoclast cultures from wildtype mice (data not shown), which would favour a mechanism in which altered coupling leads to increased osteoclast activity in this mouse model, possibly by a mechanism similar to the one observed in mutant mice with osteoblast-specific inactivation of β-catenin which results in increased bone resorption [60]. In addition to its role as a bone formation marker, the osteoblast-specific protein osteocalcin has been shown to regulate glucose metabolism and fat mass in mice [61]. Whether the elevated concentrations of osteocalcin in Lrp4ECD mice are also associated with an altered glucose metabolism will have to be determined.

The current study cannot distinguish whether the observed bone phenotype of the Lrp4ECD mutant is due to an impaired interaction of osteoblast expressed Lrp4 with Dkk1, with sclerostin, or most likely with both proteins. Future studies will have to address to what degree the phenotype is synthetic, e.g. by analyzing mice with osteoblast-specific inactivation of Lrp4 in combination with mutations in Dkk1 and sclerostin.

In conclusion, the expression of Lrp4 by osteoblasts described here adds another player to the long list of established factors that modulate canonical Wnt signaling in bone. By demonstrating that in addition to Wise, Lrp4 is able to interact with two additional important modulators of Wnt and BMP signaling, our perspective of the complexity of the integration of BMP and Wnt signaling pathways on the osteoblast surface has expanded further. Extensive further studies are clearly necessary to fully understand the mechanisms by which Lrp4 deficiency leads to impaired bone growth, increased bone turnover and polysyndactly in these mutant mice. Nevertheless the recently described association of both the SOST and LRP4 genes with BMD in humans, together with our findings suggest that LRP4 plays a physiologically important role in the skeletal development and bone metabolism not only in rodents, but in humans as well.

## References

[1] McClung MR (2005). The relationship between bone mineral density and fracture risk.. Curr Osteoporos Rep.

[2] Kanis JA, Oden A, Johnell O, Johansson H, De Laet C (2007). The use of clinical risk factors enhances the performance of BMD in the prediction of hip and osteoporotic fractures in men and women.. Osteoporos Int.

[3] Deal C (2009). Potential new drug targets for osteoporosis.. Nat Clin Pract Rheumatol.

[4] Hoeppner LH, Secreto FJ, Westendorf JJ (2009). Wnt signaling as a therapeutic target for bone diseases.. Expert Opin Ther Targets.

[5] Baron R, Rawadi G (2007). Targeting the Wnt/beta-catenin pathway to regulate bone formation in the adult skeleton.. Endocrinology.

[6] Piters E, Boudin E, Van Hul W (2008). Wnt signaling: a win for bone.. Arch Biochem Biophys.

[7] Styrkarsdottir U, Halldorsson BV, Gretarsdottir S, Gudbjartsson DF, Walters GB (2009). New sequence variants associated with bone mineral density.. Nat Genet.

[8] Styrkarsdottir U, Halldorsson BV, Gretarsdottir S, Gudbjartsson DF, Walters GB (2008). Multiple genetic loci for bone mineral density and fractures.. N Engl J Med.

[9] Kusu N, Laurikkala J, Imanishi M, Usui H, Konishi M (2003). Sclerostin is a novel secreted osteoclast-derived bone morphogenetic protein antagonist with unique ligand specificity.. J Biol Chem.

[10] Winkler DG, Sutherland MK, Geoghegan JC, Yu C, Hayes T (2003). Osteocyte control of bone formation via sclerostin, a novel BMP antagonist.. EMBO J.

[11] Winkler DG, Sutherland MS, Ojala E, Turcott E, Geoghegan JC (2005). Sclerostin inhibition of Wnt-3a-induced C3H10T1/2 cell differentiation is indirect and mediated by bone morphogenetic proteins.. J Biol Chem.

[12] Li X, Zhang Y, Kang H, Liu W, Liu P (2005). Sclerostin binds to LRP5/6 and antagonizes canonical Wnt signaling.. J Biol Chem.

[13] Ohazama A, Johnson EB, Ota MS, Choi HY, Porntaveetus T (2008). Lrp4 modulates extracellular integration of cell signaling pathways in development.. PLoS One.

[14] Willnow TE, Nykjaer A, Herz J (1999). Lipoprotein receptors: new roles for ancient proteins.. Nat Cell Biol.

[15] Strickland DK, Gonias SL, Argraves WS (2002). Diverse roles for the LDL receptor family.. Trends Endocrinol Metab.

[16] Herz J, Chen Y, Masiulis I, Zhou L (2009). Expanding functions of lipoprotein receptors.. J Lipid Res.

[17] Tomita Y, Kim DH, Magoori K, Fujino T, Yamamoto TT (1998). A novel low-density lipoprotein receptor-related protein with type II membrane protein-like structure is abundant in heart.. J Biochem.

[18] Johnson EB, Hammer RE, Herz J (2005). Abnormal development of the apical ectodermal ridge and polysyndactyly in Megf7-deficient mice.. Hum Mol Genet.

[19] Yamaguchi YL, Tanaka SS, Kasa M, Yasuda K, Tam PP (2006). Expression of low density lipoprotein receptor-related protein 4 (Lrp4) gene in the mouse germ cells.. Gene Expr Patterns.

[20] Weatherbee SD, Anderson KV, Niswander LA (2006). LDL-receptor-related protein 4 is crucial for formation of the neuromuscular junction.. Development.

[21] Zhang B, Luo S, Wang Q, Suzuki T, Xiong WC (2008). LRP4 serves as a coreceptor of agrin.. Neuron.

[22] Kim N, Stiegler AL, Cameron TO, Hallock PT, Gomez AM (2008). Lrp4 is a receptor for Agrin and forms a complex with MuSK.. Cell.

[23] Simon-Chazottes D, Tutois S, Kuehn M, Evans M, Bourgade F (2006). Mutations in the gene encoding the low-density lipoprotein receptor LRP4 cause abnormal limb development in the mouse.. Genomics.

[24] Drogemuller C, Leeb T, Harlizius B, Tammen I, Distl O (2007). Congenital syndactyly in cattle: four novel mutations in the low density lipoprotein receptor-related protein 4 gene (LRP4).. BMC Genet.

[25] Balemans W, Van Hul W (2007). The genetics of low-density lipoprotein receptor-related protein 5 in bone: a story of extremes.. Endocrinology.

[26] Williams BO, Insogna KL (2009). Where Wnts went: the exploding field of Lrp5 and Lrp6 signaling in bone.. J Bone Miner Res.

[27] Ellies DL, Viviano B, McCarthy J, Rey JP, Itasaki N (2006). Bone density ligand, Sclerostin, directly interacts with LRP5 but not LRP5G171V to modulate Wnt activity.. J Bone Miner Res.

[28] Balemans W, Ebeling M, Patel N, Van Hul E, Olson P (2001). Increased bone density in sclerosteosis is due to the deficiency of a novel secreted protein (SOST).. Hum Mol Genet.

[29] Brunkow ME, Gardner JC, Van Ness J, Paeper BW, Kovacevich BR (2001). Bone dysplasia sclerosteosis results from loss of the SOST gene product, a novel cystine knot-containing protein.. Am J Hum Genet.

[30] Balemans W, Van Den EJ, Freire Paes-Alves A, Dikkers FG, Willems PJ (1999). Localization of the gene for sclerosteosis to the van Buchem disease-gene region on chromosome 17q12-q21.. Am J Hum Genet.

[31] Balemans W, Patel N, Ebeling M, Van Hul E, Wuyts W (2002). Identification of a 52 kb deletion downstream of the SOST gene in patients with van Buchem disease.. J Med Genet.

[32] Loots GG, Kneissel M, Keller H, Baptist M, Chang J (2005). Genomic deletion of a long-range bone enhancer misregulates sclerostin in Van Buchem disease.. Genome Res.

[33] Li X, Ominsky MS, Niu QT, Sun N, Daugherty B (2008). Targeted deletion of the sclerostin gene in mice results in increased bone formation and bone strength.. J Bone Miner Res.

[34] Mao B, Wu W, Li Y, Hoppe D, Stannek P (2001). LDL-receptor-related protein 6 is a receptor for Dickkopf proteins.. Nature.

[35] Bafico A, Liu G, Yaniv A, Gazit A, Aaronson SA (2001). Novel mechanism of Wnt signalling inhibition mediated by Dickkopf-1 interaction with LRP6/Arrow.. Nat Cell Biol.

[36] Bhat BM, Allen KM, Liu W, Graham J, Morales A (2007). Structure-based mutation analysis shows the importance of LRP5 beta-propeller 1 in modulating Dkk1-mediated inhibition of Wnt signaling.. Gene.

[37] Balemans W, Devogelaer JP, Cleiren E, Piters E, Caussin E (2007). Novel LRP5 missense mutation in a patient with a high bone mass phenotype results in decreased DKK1-mediated inhibition of Wnt signaling.. J Bone Miner Res.

[38] Mao B, Wu W, Davidson G, Marhold J, Li M (2002). Kremen proteins are Dickkopf receptors that regulate Wnt/beta-catenin signalling.. Nature.

[39] Wang K, Zhang Y, Li X, Chen L, Wang H (2008). Characterization of the Kremen-binding site on Dkk1 and elucidation of the role of Kremen in Dkk-mediated Wnt antagonism.. J Biol Chem.

[40] Mukhopadhyay M, Shtrom S, Rodriguez-Esteban C, Chen L, Tsukui T (2001). Dickkopf1 is required for embryonic head induction and limb morphogenesis in the mouse.. Dev Cell.

[41] MacDonald BT, Adamska M, Meisler MH (2004). Hypomorphic expression of Dkk1 in the doubleridge mouse: dose dependence and compensatory interactions with Lrp6.. Development.

[42] MacDonald BT, Joiner DM, Oyserman SM, Sharma P, Goldstein SA (2007). Bone mass is inversely proportional to Dkk1 levels in mice.. Bone.

[43] Ai M, Holmen SL, Van Hul W, Williams BO, Warman ML (2005). Reduced affinity to and inhibition by DKK1 form a common mechanism by which high bone mass-associated missense mutations in LRP5 affect canonical Wnt signaling.. Mol Cell Biol.

[44] Boyden LM, Mao J, Belsky J, Mitzner L, Farhi A (2002). High bone density due to a mutation in LDL-receptor-related protein 5.. N Engl J Med.

[45] Balemans W, Piters E, Cleiren E, Ai M, Van Wesenbeeck L (2008). The binding between sclerostin and LRP5 is altered by DKK1 and by high-bone mass LRP5 mutations.. Calcif Tissue Int.

[46] Grotewold L, Ruther U (2002). The Wnt antagonist Dickkopf-1 is regulated by Bmp signaling and c-Jun and modulates programmed cell death.. EMBO J.

[47] Uitterlinden AG, Arp PP, Paeper BW, Charmley P, Proll S (2004). Polymorphisms in the sclerosteosis/van Buchem disease gene (SOST) region are associated with bone-mineral density in elderly whites.. Am J Hum Genet.

[48] Sims AM, Shephard N, Carter K, Doan T, Dowling A (2008). Genetic analyses in a sample of individuals with high or low BMD shows association with multiple Wnt pathway genes.. J Bone Miner Res.

[49] Kamiya N, Ye L, Kobayashi T, Mochida Y, Yamauchi M (2008). BMP signaling negatively regulates bone mass through sclerostin by inhibiting the canonical Wnt pathway.. Development.

[50] van Bezooijen RL, Svensson JP, Eefting D, Visser A, van der HG (2007). Wnt but not BMP signaling is involved in the inhibitory action of sclerostin on BMP-stimulated bone formation.. J Bone Miner Res.

[51] Willnow TE, Sheng Z, Ishibashi S, Herz J (1994). Inhibition of hepatic chylomicron remnant uptake by gene transfer of a receptor antagonist.. Science.

[52] Ducy P, Amling M, Takeda S, Priemel M, Schilling AF (2000). Leptin inhibits bone formation through a hypothalamic relay: a central control of bone mass.. Cell.

[53] Chen M, Zhu M, Awad H, Li TF, Sheu TJ (2008). Inhibition of beta-catenin signaling causes defects in postnatal cartilage development.. J Cell Sci.

[54] Nagayama M, Iwamoto M, Hargett A, Kamiya N, Tamamura Y (2008). Wnt/beta-catenin signaling regulates cranial base development and growth.. J Dent Res.

[55] Kobayashi T, Lyons KM, McMahon AP, Kronenberg HM (2005). BMP signaling stimulates cellular differentiation at multiple steps during cartilage development.. Proc Natl Acad Sci U S A.

[56] Perry MJ, McDougall KE, Hou SC, Tobias JH (2008). Impaired growth plate function in bmp-6 null mice.. Bone.

[57] Boucher P, Gotthardt M, Li WP, Anderson RG, Herz J (2003). LRP: role in vascular wall integrity and protection from atherosclerosis.. Science.

[58] Boucher P, Li WP, Matz RL, Takayama Y, Auwerx J (2007). LRP1 functions as an atheroprotective integrator of TGFbeta and PDFG signals in the vascular wall: implications for Marfan syndrome.. PLoS One.

[59] Zhou L, Takayama Y, Boucher P, Tallquist MD, Herz J (2009). LRP1 regulates architecture of the vascular wall by controlling PDGFRβ-dependent phosphatidylinositol 3-kinase activation.. PLoS One.

[60] Glass DA, Bialek P, Ahn JD, Starbuck M, Patel MS (2005). Canonical Wnt signaling in differentiated osteoblasts controls osteoclast differentiation.. Dev Cell.

[61] Lee NK, Sowa H, Hinoi E, Ferron M, Ahn JD (2007). Endocrine regulation of energy metabolism by the skeleton.. Cell.

